# The use of growth standards and corrective formulae to calculate the height loss caused by idiopathic scoliosis

**DOI:** 10.1186/s13013-016-0068-9

**Published:** 2016-02-26

**Authors:** Adrian Gardner, Anna Price, Fiona Berryman, Paul Pynsent

**Affiliations:** The Royal Orthopaedic Hospital NHS Foundation Trust, Bristol Road South, Northfield, Birmingham, B31 2AP UK; School of Clinical and Experimental Medicine, University of Birmingham, Edgbaston, Birmingham, B15 2TT UK

**Keywords:** Scoliosis, Idiopathic, Height, Loss, Formula

## Abstract

**Background:**

Loss of trunk height caused by scoliosis has been previously assessed using different mathematical formulae. However, these are of differing algebraic construction and will give a range of values for the same size of scoliosis curve. As such, the following study attempted to determine the most valid published formulae for calculating height loss caused by idiopathic scoliosis based on reported growth charts.

**Methods:**

The height and sitting height for a group with idiopathic scoliosis were measured. These were plotted on published growth standards. The size of the coronal curves and the thoracic kyphosis was measured. Height was corrected for the size of the scoliosis using the formulae and replotted on the growth standards. The data spread on the standard was analysed for significant differences between the median and the 5th or 95th centile, and between data outside the 5th and 95th centile.

**Results:**

The sitting to standing height ratio growth standard was used in the analysis as it minimised errors across the different growth standards, given that these standards come from different original populations. In the female group significant differences in the data spread were seen using the formulae of Bjure, Ylikoski and Hwang. Non-significant results were seen for the Kono and Stokes formulae. All formulae caused no significant differences in data spread across the growth standard in the males group.

**Conclusions:**

When assessing against growth standards, the formulae of Kono and Stokes are the most valid at determining height loss caused by idiopathic scoliosis.

## Background

Idiopathic scoliosis (IS) is of unknown origin and includes scoliosis seen in the adolescent, between the ages of 10 and 18, and in early adult life once older than 18. It is a growth related deformity of the spine. The deformity leads to a loss of standing height and it is common for surgeons to be asked how much height will be regained when a patient undergoes a corrective scoliosis fusion procedure. Whilst the ‘height gained’ during surgery is dependent on many factors that cannot reliably be predicted pre-operatively, it is possible to calculate the height that has been lost through formulae that have been published [1–5]. All of the formulae have a different mathematical construction and the aim of this paper is to identify which formula gives the most valid estimation of scoliosis related height loss. This will be performed through an assessment of the concurrent validity of the different formulae with reference to previously published cross-sectional growth standards.

## Methods

The standing and sitting heights of a group of patients with IS were measured. All had one curve of at least 10° in the coronal plane to satisfy the definition of structural scoliosis [6] even if the curve pattern was of more than one curve. None of the group had undergone surgical intervention. The measurements were performed with calibrated stadiometers by two research nurses who were not involved in the analysis of the data. The sitting height to standing height ratio was calculated. The measurements were all taken at the same time of day to eliminate the change in height that can occur as the day passes [7]. Participants were asked to stand in an ‘upright but natural’ posture so that the position of the body during height measurement matched the body position during the subsequent radiograph.

The measured data were plotted on the published growth standard. The World Health Organisation (WHO) standard for standing height was used with the data sub divided by sex [8]. This was repeated for sitting height on the Danish sitting height standard and for sitting to standing height ratio, again on the Danish standard [9]. The WHO standing height standard is presented as the median value ± 2 z-scores [8], whereas the Danish sitting height and sitting to standing height ratio is presented as a median value with 5th and 95th centiles [9].

The size of the coronal scoliosis curves and the sagittal kyphosis and lordosis was measured from standing whole spine radiographs accessed digitally. The radiographs were all taken in the same standardised fashion with the sagittal radiograph taken with the arms in the ‘fists on clavicle’ position to eliminate the effect of arm position on overall sagittal alignment [10].

All coronal curves were measured between the most angled end plates as per the Cobb method [11]. The kyphosis was measured between the superior endplate of T1 and inferior endplate of T12. The lordosis was measured between the superior endplates of L1 and S1. Radiographic measurements were made by an experienced scoliosis surgeon not involved in the measurement of the patient’s height. If there was no curve in a part of the spine this was recorded as 0° for that particular segment.

**Fig. 1:**
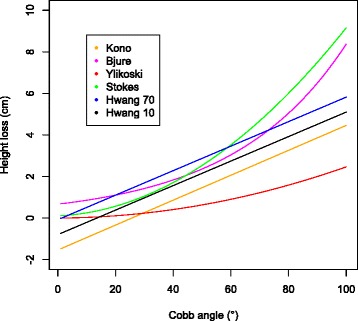
Height loss formulae plotted as mathematical functions for a single curve pattern. Note: The Hwang formula is depicted as two parallel lines, Hwang 10 and Hwang 70. This allows for a measure of kyphosis as required by the formula. A kyphosis of between 10° and 70° will include all of the data presented in this series which would be found between the two lines

The formulae for calculating height loss are shown mathematically in Table 1 and graphically in Fig. 1. The height loss caused by the scoliosis was calculated using published formulae [1–5] for each participant. The height loss was then added to the standing or sitting height. The new ‘corrected’ heights were then replotted on the appropriate standard. The sitting to standing height ratio was the most appropriate standard to measure against as it does not include absolute values and thus any bias caused by a standard of a particularly tall population will be minimised. The sitting to standing height ratio is calculated by dividing the sitting height by the standing height.

**Table 1:** Formulae for height loss caused by the scoliosis (y equalling height loss in cms). The Bjure formula, which is logarithmic, has been changed to a form equivalent to the other formulae for clarity

Name of formula	Formula	Description of formula
Bjure	y = 10 ^0.011x – 0.177^	x is Cobb angle of the major curve
Kono	y = (0.6 (x - 30) + 2.6)/10	x is combined Cobb of all curves
Ylikoski	y = (0.0062 x + 0.0024 x^2^)/10	x is combined Cobb of all curves
Stokes	y = (1 + 0.0066 x + 0.0084 x^2^)/10	x is mean Cobb of the largest two curves
Hwang	y = 0.059 x + 0.012z – 0.919	x is the major thoracic Cobb, z is T5 to T12 kyphosis

Two different assessments were made to assess which formulae were most valid for calculating the height loss due to the scoliosis. The first method counted the number of data points on either side of the median but between ± 2 z-scores or between the 5th and 95th centile (from here on defined as the inner data spread) and compared them. Second the number of data points above the + 2 z-scores or the 95th centile were compared with the number of data points below – 2 z scores or the 5th centile (defined as the outer data spread). A statistical comparison was made using a test of equal or given proportions [12]. With data added to the standard, the ‘best data fit’ would have no significant difference when comparing the inner data spread either side of the median or outer data spread outside ± 2 z-scores or the 5th and 95th centiles. Thus the formula that gives a non-significant result by both analyses would be the most valid available for assessing the height loss from the scoliosis preoperatively. Significant results were defined as a *p* value ≤ 0.05.

In all of these formulae, *y* is the calculated height lost and *x* is the Cobb angle or sum of Cobb angles. In the Hwang [2] formula, *z* is the kyphosis angle. As not all radiographic series included a sagittal radiograph concordant with the time of height measurement that could be measured for thoracic kyphosis, the total number of data points in the Hwang formula calculation was reduced accordingly.

Bland Altman analysis [13] was also performed. This compared the results of the formulae against each other to calculate the mean and 95 % limits of agreement for the differences between the formulae.

All statistical and graphical analysis was performed using R [14]. The patients in this cohort have been followed up for a minimum of 24 months as part of their medical care although follow up is not part of this paper.

## Results

In the scoliosis group, there were 161 females and 44 males. In the analysis for the Hwang formula there were 137 females and 37 males. Tables 2 and 3 show the demographics of the groups analysed.

**Table 2:** Demographics of the study group

	Mean age	SD of age (months)	Age range (months)
Males	15 years 7 months	21	11 years 7 months to 20 years and 3 months
Females	15 years 1 month	30.5	8 years and 4 months to 27 years and 11 months

**Table 3:** Size of scoliosis measured in the study group

	Mean curve size (°)	SD of curve size (°)	Range of curve size (°)
Males	38	22	0–81
Females	44	21	2–96

**Fig. 2:**
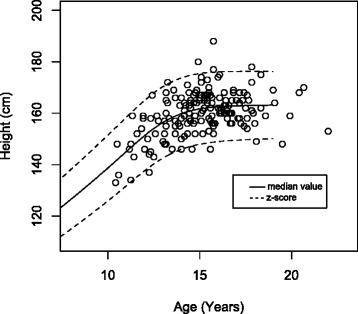
Standing height of the female group plotted on the WHO standard for standing height

**Fig. 3:**
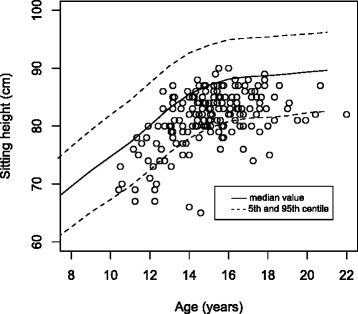
Sitting height of the female group plotted on the Danish sitting height standard

**Fig. 4:**
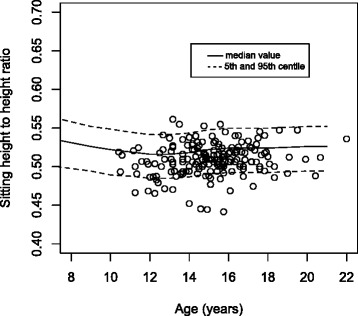
Sitting height to standing height ratio of the female group plotted on the Danish ratio standard

When standing height was plotted on the WHO height standards there was no significant difference between the inner or outer data spread for both the male and female groups (see Fig. 2). A plot of sitting height of the scoliotic females on the Danish sitting height standard shows that the scoliotic females have a lower sitting height than the standard (Fig. 3) [9]. There were significantly more data points between the median and the 5th centile compared to those between the median and the 95th centile (*p* < 0.01), representing an unequal inner data spread. Similarly, there was a significant difference in the number of outliers below the 5th compared to above the 95th centile (*p* < 0.01), an uneven outer data spread. The ratio of sitting height to standing height was also plotted on the Danish standard [9]. Although pictorially the data looks less shifted compared to the sitting height data, again a significant difference in the inner data spread (*p* < 0.01) and outer data spread (*p* < 0.01) for females was seen (Fig. 4). This is repeated, although it is less striking, with the male group, with a significant difference seen in sitting height for both inner (*p* < 0.01) and outer (*p* < 0.01) data spread. For the sitting height to standing height ratio both the inner data spread (*p* < 0.01) and outer data spread (*p* < 0.01) were significant.

Corrected standing and sitting height and corrected sitting height to standing height ratios were calculated. This was done by adding the calculated height loss using the different formulae to the original measured data and then replotting on the appropriate standards. For the Stokes method, only the formula for double curves was used, averaging the size of the two curves as in this series there were not enough truly single curves to make analysis of this group meaningful [4].

**Fig. 5:**
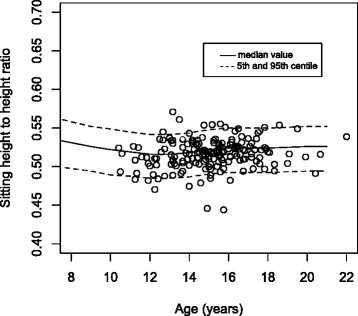
Female sitting height to standing height ratio corrected with the Ylikoski formula

The table of results (Table 4) shows that for the female group, for the inner data spread, all formulae other than the Kono et al. [3] and Stokes [4] formula caused a significant result, whereas for the outer data spread none of the formulae caused a significant difference in data spread (see Fig. 5). For males, no formulae caused a significant result in data spread for either the inner and outer data spread.

**Table 4:** A table of the spread of the inner and outer data spread for males and females

Females	Above 95th	Below 5th	Significance (*p* value)	Between median and +95th	Between median and +5th	Significance (*p* value)	Total
Standing height	5	5	1	76	75	1	161
Sitting height	0	36	<0.001*	16	109	<0.001*	161
SH/H ratio	4	24	<0.001*	43	90	<0.001*	161
Standing Bjure	11	3	0.056	91	56	<0.001*	161
Standing Ykilowski	11	2	0.056	90	58	<0.001*	161
Standing Kono	16	2	0.002	98	45	<0.001*	161
Standing Stokes average	19	1	<0.001*	103	38	<0.001*	161
Standing Hwang	7	3	0.334	78	49	<0.001*	137
Sitting Bjure	0	14	<0.001*	49	98	<0.001*	161
Sitting Ylikowski	0	15	<0.001*	52	94	<0.001*	161
Sitting Kono	2	8	0.108	86	65	0.026	161
Sitting Stokes average	3	6	0.498	89	63	0.002	161
Sitting Hwang	0	12	0.001*	32	93	<0.001*	137
Ratio Bjure	10	13	0.665	49	138	<0.001*	161
Ratio Ylikowski	9	12	0.652	53	87	<0.001*	161
Ratio Kono	13	6	0.156	64	78	0.145	161
Ratio Stokes average	11	6	0.319	79	65	0.145	161
Ratio Hwang	9	11	0.816	39	78	<0.001*	137
Males	Above 95th	Below 5th	Significance (*p* value)	Between median and +95th	Between median and +5th	Significance (*p* value)	Total
Standing height	1	3	0.6088	23	17	0.2844	44
Sitting height	1	10	0.01*	4	29	<0.001*	44
SH/H ratio	0	8	0.009*	11	25	0.005*	44
Standing Bjure	2	2	1	27	13	0.005*	44
Standing Ykilowski	2	2	1	27	13	0.005*	44
Standing Kono	2	2	1	29	11	<0.001*	44
Standing Stokes average	2	2	1	30	10	<0.001*	44
Standing Hwang	1	2	1	24	10	0.002*	37
Sitting Bjure	1	5	0.205	11	27	0.001*	44
Sitting Ylikowski	1	5	205	10	28	<0.001*	44
Sitting Kono	1	2	1	16	25	0.087	44
Sitting Stokes average	1	2	1	22	23	1	44
Sitting Hwang	1	4	0.354	8	24	<0.001*	37
Ratio Bjure	0	4	0.125	16	24	0.134	44
Ratio Ylikowski	0	5	0.065	16	23	0.198	44
Ratio Kono	1	5	0.205	21	17	0.519	44
Ratio Stokes average	2	5	0.431	22	16	0.282	44
Ratio Hwang	0	4	0.123	15	18	0.64	37

Significant results marked with *

Bland Altman analysis showed that the mean difference between all formulae was less than 3 cm (2.97 cm) in both males and females with 95 % limits of agreement no greater than 5.52 cm [13].

## Discussion

**Fig. 6:**
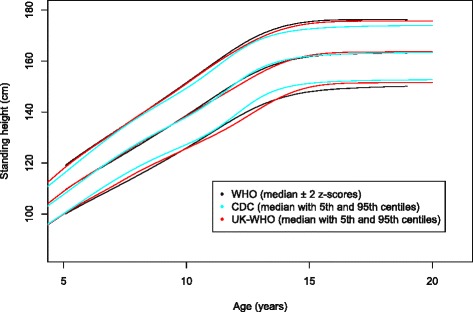
A plot comparing the growth standards for standing height for WHO [7], CDC [15] and UK-WHO [16] for females

This is the first time that growth standards have been used to reference height loss and corrected height loss in scoliosis to our knowledge. The World Health Organisation (WHO) standing height standards [15] were chosen over those of the Centre for Disease Control (CDC) [16] because, for the over 5 year olds, the data is the same for UK specific standards known as the UK-WHO [17] and would be the most appropriate to the widest worldwide audience. When assessing the growth standards for clinical relevance to this topic in this age group, there is very little difference between any of the standards (Fig. 6) [18].

A sitting height standard is not published by the WHO but the Danish standard used here is easily accessible [9]. The Danish standard also includes growth standards for leg length and sitting height to standing height ratios. It is acknowledged that the Danish are generally an exceptionally tall race and thus placing UK sitting height data onto a Danish standard may generate a false impression of the data [19]. The use of a sitting to standing height ratio eliminates this problem as the absolute value of height is removed from the calculation. Thus, assuming that the ratio of sitting to standing heights is within a similar range between the Danish and UK populations (which given the geographical and historical pasts of both countries is felt to be a reasonable assumption), then the use of this standard is also reasonable. The sitting height standard and sitting height to standing height ratio are presented as median and centiles with the 5th and 95th centile shown here [9]. There is a slight difference between the ± 2 z-scores and 5th and 95th centiles. Two standard deviations are equivalent to 95.45 % of data from the mean in a normal distribution whereas only 90 % is covered between median and 5th or 95th centile. In the setting of this study this difference was not felt to be clinically relevant. The data here demonstrates that there is trunk height loss caused by the presence of a scoliosis as seen on sitting height and sitting height to standing height ratio growth standards. All of these growth standards were created from large numbers of participants minimizing the effects of different populations and outliers to give an accurate description of growth for both standing and sitting height.

In clinical practice, patients commonly ask about the height that will be regained following scoliosis surgery. It is very difficult to estimate this pre-operatively as the exact end result of scoliosis correction is dependent on intraoperative factors. The best that can be done is to estimate the amount of height loss secondary to the presence of the scoliosis. This can be achieved pre-operatively using a variety of published formulae that have been detailed here [1–5]. Post-operatively it is also possible to calculate rather than measure the height gained following a scoliosis correction through the use of formulae published by Watanabe and Hosagane [20], Spencer et al. [21] and Sarlak et al. [22] using both pre-operative and intra-operative criteria but these calculation methods are only possible after the event.

The first attempt to calculate height loss caused by a scoliosis was by Bjure et al. [1]. They developed their formula as a way of finding the true height of the thorax in the absence of deformity for the assessment of respiratory function in those with scoliosis. The formula was originally published in 1968 [1] with a typographic error and the formula was corrected to the one used in this paper in 1970 [23]. The weakness of their formula is that it only took into account the major curve in the coronal plane and no assessment was made of the sagittal plane. Further criticism has been voiced questioning the accuracy of a logarithmic scale where errors will be greater with a larger Cobb angle, and also with concerns over the lack of standardisation of radiographs for magnification errors [3].

A new formula was proposed by Kono et al. [3] in 2000. They reviewed 140 scoliosis radiographs with both single and double curve patterns, and calculated the true length of the spine in the anteroposterior plane (AP) looking to improve on the Bjure formulae for respiratory function assessment. The calculated loss of height was then analysed with the size of the Cobb angle. The conclusion of the paper was that their new formula should be used instead of the Bjure formula as all of the curves contributed to the height loss rather than just the major curve. However, again, this formula did not take in to account the effect of the sagittal plane and changes in thoracic kyphosis seen with growth and deformity.

In 2003, Ylikoski [5] measured the height of 1500 Finnish girls with scoliosis greater than 10° and compared this to the standing height of the average non-scoliotic girl in Finland. The height loss caused by the scoliosis was measured in the AP plane by subtracting the direct distance between the upper endplate of the T4 vertebral body and the lower endplate of the L4 vertebral body from the measured distance using a flexible wire through all of the vertebral bodies from T4 to L4. The thoracic kyphosis was measured in the same way between the upper endplate of T4 and the lower endplate at the distal end of the kyphosis most commonly seen at T12. A normal value of 29° of thoracic kyphosis was taken from previous work. In his analysis of the first 30 patients, kyphosis greater than 29° led to an addition and less than 29° a subtraction from the overall end height. The conclusion stated that the amount added or subtracted due to thoracic kyphosis did not affect the height of patients with scoliosis when compared to the heights of girls with a normal kyphosis of the same age in a non-scoliotic group; the sagittal plane was therefore excluded from the final formula. There was no assessment of lumbar lordosis for this formula and the effects of the lumbar levels were ignored. In addition, this formula, if applied strictly, is only applicable to females, having been constructed from a female group.

Stokes [4] published a retrospective radiographic review of 387 patients with adolescent or juvenile scoliosis between 9 and 20 years of age comprising 182 single curves and 205 double curves. The size of the scoliotic curves was measured. Spinal length was calculated through the addition of the heights of the vertebral bodies and discs between T5 and L5 from previously stored three dimensional coordinate data of the position of the spine in space. Spinal height was measured from standardised radiographs. Height loss was defined as the difference between spinal height and spinal length. Analysis of height loss with the degree of spinal curvature led to the development of formulae for single and double curve patterns. Stokes [4] stated that it would be appropriate to include the compensatory curve in the calculation of height loss even if it was not structural by averaging the two Cobb measurements. This analysis again only looked at the coronal and not the sagittal plane.

Hwang et al. [2] retrospectively looked at a group of 447 patients with Lenke types 1, 2, and 3 curves in both males and females having undergone only posterior scoliosis procedures. Their formula concluded that height gained is related to the amount of coronal curve and the size of the kyphosis in the thoracic spine. The authors accept that this formula only explains some of the height loss secondary to a scoliosis as it is derived from the post-operatively height. It is likely that this is because a deformity correction is never 100 % and the post-operative spine will not represent the true non-deformed spinal height [24].

The Hwang formula [2] is the only formula to include an assessment of the three dimensional nature of a scoliotic deformity in the calculated height loss. It is known that IS is a lordotic deformity in the sagittal plane, thus there will be less thoracic spine kyphosis when compared to population norms. This may well result in an addition to vertical height rather than the subtraction caused by the coronal plane deformity [25, 26]. Kyphosis in the Hwang [2] paper was measured using the Cobb method between T2 and T12. In this paper, the Hwang formula has been used with the size of the main thoracic curve interpreted as the ‘major thoracic Cobb’ [2]. The proximal thoracic curve is not included as in the Hwang paper's multivariate analysis, thoracic curve magnitude is quoted rather than proximal or main thoracic curve magnitude [2]. It can be difficult to identify whether a proximal curve is a true structural curve and for consistency only the main thoracic curve was included in this analysis.

The assessment of which of the formulae gives the most valid calculation of height loss secondary to scoliosis has been performed using a test of equal or given proportions on the spread of data points above or below the median or 50th centile line, and outside ± 2 z-scores or the 5th and 95th centile [12]. This has been defined as either the inner or outer data spread. The assumption behind this analysis is that there is an equal distribution in the growth standard at any one age point and the amount above and below the median at that age point will be equal. The formula that changes the data, from initially having a significant difference in spread to being non-significant around the median or as outliers on a growth standard will therefore represent the formula which gives the most valid calculation of height loss. This analysis has been performed on the sitting height to standing height ratio standard to eliminate any effects of the difference in total height between the Danish and UK population.

The uniform spread of data points across the WHO standing height standard, even with a loss of height caused by a scoliosis, demonstrates that the spread of data between ± 2 standard deviations from the median is too large to demonstrate the generally small changes in height caused by most scoliotic curves. This is because the loss of height caused by the scoliosis when viewed as a fraction of total body height in the standing position is small and makes little difference to the whole. In this series the mean height loss across all formulae was 3.38 cm for females and 2.86 cm for males. When expressed as a fraction of sitting height, the change is more obvious and can be seen on the sitting height standard. This then suggests that both sitting and standing height standards should be used to chart height in those with scoliosis to identify the subtle loss from the spinal curve, agreeing with the previous literature [27].

Using the definition of most valid as no significant differences in the number of data points for either the inner or outer data spread when plotted on the sitting to standing height ratio, it is seen that the Kono [3] or Stokes [4] formulae are the most valid for females. In the male group, all of the formulae by either definition are equally valid as they are not significant. As is reflected by a 10 to 1 ratio of females to males with AIS, the female group is larger than the male group. It may well be that with a larger number in the male group the results may change and be closer to those seen in the female group.

Bland-Altman analysis for all pairs of formulae allows a comparison of the differences between them [13]. In this paper, as the absolute height or length of the spine has not been measured, an analysis against ‘the truth’ is not possible. By sequentially comparing the results of one formula against all of the others the appropriate analysis can be performed. The differences seen in the Bland Altman analysis are small and it is felt would not be deemed to be of clinical significance.

Four of these formulae for calculating loss of height were compared against a measured loss of spine height on an individual basis by Tyrakowski et al. [28]. The ‘true height loss’ caused by the scoliosis was calculated as the difference between the measured vertical height of the spine between the endplates of T1 and S1 and the measured length of the spine between the endplates of T1 and S1 but through the centroids of all vertebral bodies between. This was then compared to the calculated height loss using four of the five formulae used in this paper [1, 3–5]. The authors conclude that none of the formulae agree about the height loss secondary to the scoliosis. They also note that patients with the same curve sizes but a different overall height will have the same height loss by any of the formulae, but this does not take into account the initial trunk height which may then over or under predict the individual height loss. The use of growth standards versus an individual radiograph in this paper is a different approach in defining the ‘true height’ to compare with a calculated height. The advantage of a radiograph is that the measurement is of just the spine excluding the head, neck and pelvis. The disadvantage of the radiograph method is that it is individual to that particular patient which, depending on whether the child is tall or small for age may, as described above, over or under predict height loss. Growth standards on the other hand, like the formulae themselves, represent a population. The flaws inherent in a mathematical description of a biological process will be minimized through this approach.

The assumption behind the use of growth standards in this setting is that the scoliotic spine is the same length as the non-scoliotic spine when all other variables are equal when at the same chronological age. This may be flawed as there is some evidence to suggest that scoliosis is an effect of an ‘over long’ spine or rapid early growth [29]. Further research states that scoliotic children are taller than their non-scoliotic counterparts [26] and that those with more severe curves are taller than those with smaller curves or curves secondary to a leg length discrepancy and pelvic tilt [25]. One hypothesis suggests that this effect may represent the uncoiling of thoracic kyphosis which is greater in a large scoliosis or a different pattern of growth and growth velocity [25, 29–32]. The only way to be absolutely sure would be to measure the length of the spine in three dimensions, possibly from an MRI scan or using an EOS three dimensional scanner, and relate this length to the growth standards and confirm the validity of these formulae against a measured true spinal length.

## Conclusion

The height loss seen in the presence of scoliosis is best documented on sitting height and sitting to standing height ratio growth charts. This height loss can be calculated pre-operatively and the most valid result will be obtained with the formulae described by Kono et al. [3] or Stokes [4] when compared to cross-sectional growth standards.

### Research ethics committee approval

Ethical approval from the Research Ethics Committee has been obtained for this study reference 15/EM/0283.
