# Erratum to: The use of growth standards and corrective formulae to calculate the height loss caused by idiopathic scoliosis

**DOI:** 10.1186/s13013-016-0078-7

**Published:** 2016-07-20

**Authors:** Adrian Gardner, Anna Price, Fiona Berryman, Paul Pynsent

**Affiliations:** The Royal Orthopaedic Hospital NHS Foundation Trust, Bristol Road South, Northfield, Birmingham, B31 2AP UK; School of Clinical and Experimental Medicine, University of Birmingham, Edgbaston, Birmingham, B15 2TT UK

## Erratum

After publication of this article [1] the author brought to our attention that the formula of Stokes in Table 1 is incorrect. The correct formula is y = (1 + 0.066x + 0.0084x^2^)/10 where x represents the mean Cobb angle of the largest two curves in the scoliosis and y the height loss in centimetres.
